# Variation of Lower-Limb Muscle Activation Asymmetry in Step Incremental and Constant-Power Pedaling Exercise

**DOI:** 10.3390/s26020587

**Published:** 2026-01-15

**Authors:** Seyed Hamidreza Heidary, Reza Ahmadi, Shahram Rasoulian, Samira Fazeli Veisari, David Auslander, Saied Jalal Aboodarda, Amin Komeili

**Affiliations:** 1 Department of Mechanical and Manufacturing Engineering, University of Calgary, Calgary, AB T2N1N4, Canada; seyedhamidreza.heida@ucalgary.ca (S.H.H.); reza.ahmadi3@ucalgary.ca (R.A.); 2 Faculty of Kinesiology, University of Calgary, Calgary, AB T2N1N4, Canada; shahram.rasoulian@ucalgary.ca (S.R.); saiedjalal.aboodarda@ucalgary.ca (S.J.A.); 3 Department of Biomedical Engineering, University of Calgary, Calgary, AB T2N1N4, Canada; samira.fazeliveisari@ucalgary.ca; 4 Mechanical Engineering Department, University of California, Berkeley, CA 94720, USA; dma@me.berkeley.edu

**Keywords:** asymmetry, cycling biomechanics, muscle activity, exercise intensity, normalized symmetry index

## Abstract

Asymmetry, defined as unequal neuromuscular activation or mechanical performance between contralateral limbs, plays a critical role in cycling efficiency and injury risk. While kinematic and kinetic measures are commonly used to assess asymmetry, surface electromyography (EMG) signals offer an additional perspective on neuromuscular asymmetry. This study evaluated muscle activation asymmetry during cycling using the Normalized Symmetry Index (NSI), a metric that quantifies differences in kinematics and kinetics between limbs, where higher values indicate greater asymmetry. NSI was calculated from EMG recordings of seven lower-limb muscles under two test conditions: step incremental and constant-power cycling to task failure. Twenty recreationally active participants performed both tests on a stationary ergometer while EMG data were collected bilaterally. Step incremental cycling resulted in a significant reduction in NSI for key muscles in the quadriceps group: vastus medialis (from 44% to 21%, *p* < 0.001), vastus lateralis (from 45% to 22%, *p* = 0.002), rectus femoris (from 54% to 24%, *p* < 0.001), and biceps femoris (from 52% to 29%, *p* = 0.003). No significant changes were observed for the tibialis anterior, soleus, or gastrocnemius medialis. In contrast, under constant-power conditions, NSI values remained unchanged over time for all muscles (all *p* > 0.05), with average NSI values ranging from 12% to 30%, indicating consistent bilateral activation. These findings highlight the sensitivity of surface EMG in detecting workload-dependent changes in muscle activation asymmetry and suggest that higher cycling intensities, compared to lower ones, may promote more balanced engagement of primary cycling muscles.

## 1. Introduction

In the context of human movement (e.g., cycling and locomotion), asymmetry is defined as a deviation from mirror symmetry across the coronal axis [1]. Asymmetry analysis has been applied in the prior literature to investigate differences in lower-limb kinematics and kinetics, including parameters such as joint angles, force, and power outputs [2,3,4]. The study of asymmetry in lower limbs has received significant attention in sports and rehabilitation science [5,6,7,8]. Detection and mitigation of asymmetry can have important implications for optimizing performance [9,10,11], reducing musculoskeletal injuries, and studying joint loading imbalance, highlighting the relevance of its assessment for performance enhancement [12] and clinical rehabilitation [13]. Targeted interventions, such as unilateral strength or plyometric training, have been shown to reduce asymmetry and enhance functional performance [14]. However, asymmetry does not typically arise from a single source; instead, it results from the interaction among physiological, biomechanical, and environmental factors [15,16,17,18]. 

Recent studies highlight the value of electromyography- (EMG) based approaches for assessing inter-limb asymmetry in cycling. Bilateral EMG approaches examining muscle synergies and coactivation patterns during cycling also yield insights relevant to asymmetry evaluation [19]. Evidence from stroke rehabilitation indicates that EMG-triggered cycling can reduce inter-limb muscle activation asymmetries [20]. Bao et al. showed that bilateral EMG complexity can reveal pedaling asymmetry and clinically relevant motor deficits [21], while Iglesias-Caamaño et al. established task- and category-specific asymmetry thresholds to guide individualized training in competitive cyclists [22]. In this context, recognizing early signs of functional muscle imbalance is crucial for implementing targeted interventions that support accurate quantification and management of asymmetry.

Although cycling is often considered a bilaterally symmetric activity, studies have reported asymmetries in kinematic and kinetic parameters of the lower limbs during this mode of exercise. Researchers have employed various methods to quantify asymmetries during cycling. Common approaches include the calculation of Symmetry Indices (SI), which represent the percentage difference between values of contralateral limbs divided by their average, and the ratio of biomechanical factors (e.g., pedaling force or power output) between dominant and nondominant limbs [23,24,25]. However, these traditional indices can be highly sensitive to measurement conditions, such as values close to zero, the type of exercise performed and the intensity of cycling [25,26]. To address these challenges, alternative parameters, such as the Normalized Symmetry Index (NSI), have been introduced [27]. NSI is a quantitative index used to assess the discrepancy in the biomechanical parameters of contralateral limbs during a given movement. It compares the magnitude of biomechanical variables derived from both limbs, expressing their difference as a percentage relative to their combined average activity. A lower NSI value indicates a higher level of symmetry, while a higher value suggests a greater imbalance between the limbs [28,29,30]. NSI addresses limitations of traditional indices such as SI. More specifically, it uses a fixed, bounded scale (0–100%) that preserves true amplitude differences, enhances sensitivity to asymmetries, and enables consistent comparisons across variables and subjects [31,32]. Unlike traditional SI, which can become unstable when values approach zero, NSI solves this problem by normalizing the difference relative to the mean of both limbs. This approach minimizes singularities and ensures that asymmetrical values remain interpretable and comparable across different exercise conditions. This index enables the detection of subtle differences in lower-limb function throughout a movement, rather than relying solely on discrete peak values.

Pedal force, a critical component in asymmetry analysis, has been extensively studied using asymmetry indices. For instance, when cycling intensity increased from 50% to 85% of maximum power, bilateral asymmetry was reduced from 14.1% to 1%, suggesting that higher workloads may help reduce limb differences [33]. Similarly, research on master cyclists over 50 years old reported a significant decrease in the torque asymmetry index from 30 ± 8% to 23 ± 13% as power output increased [34]. Higher pedaling cadences also decreased power output asymmetry, which is correlated with pedal force output [35]. Despite this evidence, understanding muscle activation patterns could inform more personalized training and rehabilitation methods designed to address both mechanical and physiological imbalances [36,37,38,39,40]. However, while force- and torque-based asymmetry indices quantify the mechanical output differences between limbs, they do not directly capture the underlying neuromuscular strategies that may drive or compensate for these imbalances.

Asymmetry indices were derived from EMG muscle activation in activities such as walking [41] and sprinting [42], especially in individuals with unilateral conditions like ACL reconstruction and stroke [43,44,45,46]. Although EMG is widely used to analyze muscle activation, its application in studying functional muscle asymmetry in cycling remains limited. In addition, analyzing muscle function asymmetry under step incremental and constant-power cycling to task failure can elucidate how different exercise intensities and neuromuscular fatigue status can affect asymmetry responses.

Therefore, the objective of this study is to quantify asymmetry in muscle activity responses using the NSI parameter in lower-limb muscles during step incremental and constant-power pedaling tasks. The NSI, derived from EMG amplitude, quantifies inter-limb differences in activation magnitude on a normalized scale; however, it does not capture temporal aspects of activation (e.g., onset timing, phasing, or coordination). Thus, NSI for EMG signal will be interpreted as an amplitude-based measure of asymmetry rather than as a marker of motor coordination. The results will elaborate on whether (i) calculating NSI based on EMG data can be a sensitive method to detect asymmetry, (ii) increasing power output during step incremental test can alter NSI value, and (iii) the fatigue experienced during exhaustive constant-power cycling tasks can alter the asymmetry index. It is hypothesized that (i) NSI calculated based on EMG data can detect asymmetry, (ii) NSI value decreases with increasing power output in the step incremental test, and (iii) fatigue does not affect NSI value during the constant-power output test.

## 2. Materials and Methods

### 2.1. Participants

Twenty recreationally active males (*n* = 8) and females (*n* = 12) (22.6 ± 4.5 years, 171.8 ± 7.7 cm, 70.6 ± 12.5 kg) participated in the study. All participants completed the Physical Activity Readiness Questionnaire (PAR-Q+) and were free of any health conditions or injuries that could preclude them from performing high-intensity cycling or maximal isometric knee extensions. Participants were also instructed to avoid vigorous physical activity for two days prior to testing and to refrain from caffeine and alcohol consumption for at least 12 h beforehand. All participants provided written informed consent prior to the study, which was conducted in the Human Performance Laboratory at the University of Calgary. Ethical approval was obtained from the University of Calgary Research Ethics Board (REB #24-1803).

### 2.2. Test Protocol

**Figure 1:**
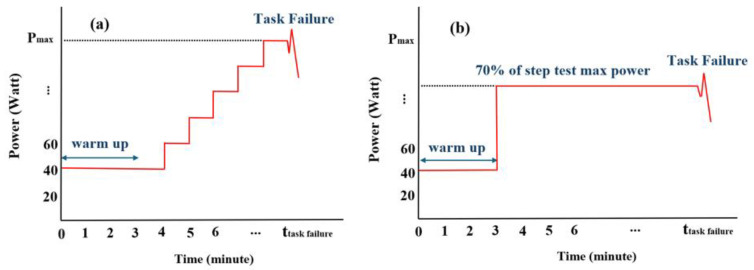
(**a**) Power progression during the step incremental test protocol, with 20 W power output increments every minute until task failure, and (**b**) power profile during the constant-power test, performed at 70% of individual peak power until task failure.

Each participant completed one step incremental and one constant-power test using a cycling ergometer (Echelon Connect EX-5, Echelon Fitness Multimedia LLC, Chattanooga, TN, USA). Pedal power was measured with dual-sided Sensix instrumented pedals. The reliability of the system was studied in a previous work [47]. At the start of each data-collection day, the pedal sensors were warmed for ~10 min and zeroed without load, followed by the manufacturer’s calibration guideline. The power output was displayed on a screen to help participants maintain the target workload. The step incremental test began with a 3-min warm-up at 40 W and 70 RPM, after which the cycling power output increased by 20 W per minute until the point when the participant could no longer maintain the required cadence or power with a variation exceeding ±20% for at least 10 s despite verbal encouragement, i.e., task failure (see Figure 1). To minimize the effect of cadence variability on NSI, the cadence was continuously monitored, and participants received verbal feedback whenever it deviated from the 65–75 RPM range. The ergometer was also equipped with an encoder (MR076G060A120N00, RLS, Komenda, Slovenia), and cadence values were double-checked using the I-Crank software to ensure that pedaling velocity remained within the specified range throughout the tests.

The constant-power test also began with a 3-min warm-up at 40 W and 70 RPM, followed by sustained cycling at 70% of the maximum power achieved in the step incremental test, continuing until task failure (see Table 1). A constant load of 70% maximal power was used in cycling fatigue and endurance studies [48,49,50]. Moreover, a constant-power test at 76% ± 3% of Pmax (determined from a step-incremental test) and 60 RPM resulted in an average time to exhaustion of 11.11 ± 1.86 min [51]. Thus, the proposed constant-power test at 70% of the maximum power and 70 RPM was deemed sufficient to induce fatigue below the maximal domain, thereby facilitating the temporal variation in NSI in EMG signal. Moreover, during the constant-power test, participants continuously reported their perceived exertion (RPE; Borg 6–20 scale), pain level (0–10), and breathing difficulty. These subjective measures were used as objective indicators to verify the onset and progression of fatigue and to confirm exhaustion at task failure [52,53,54]. A minimum 24-h rest period was provided between the two tests to prevent fatigue carryover between sessions. Saddle height and handlebar positions were standardized according to participant height to ensure consistency [55].

### 2.3. Experimental Setup

A stationary ergometer (Echelon Connect EX-5, Echelon Fitness Multimedia LLC, Chattanooga, TN, USA), equipped with instrumented pedals (Sensix, Poitiers, France; ICS-MB and Mountain-BMX, Italy; Shimano SPD, Osaka, Japan) featuring integrated 3D force sensors, was used. These sensors recorded pedal forces at a sampling rate of 250 Hz. Crank position was monitored using an incremental encoder (Model LM13, RLS, Komenda, Slovenia), which measured changes in angular displacement relative to a reference point. Additionally, two embedded pedal encoders measured crank-pedal angles, enabling the transformation of pedal force components into the global coordinate system. Participants wore cycling shoes of appropriate sizes, with calibrated cleats aligned to the pedals; cleat positioning was verified using cardboard shoe insoles to ensure consistency.

### 2.4. Electromyography Data Acquisition and Processing

**Figure 2:**
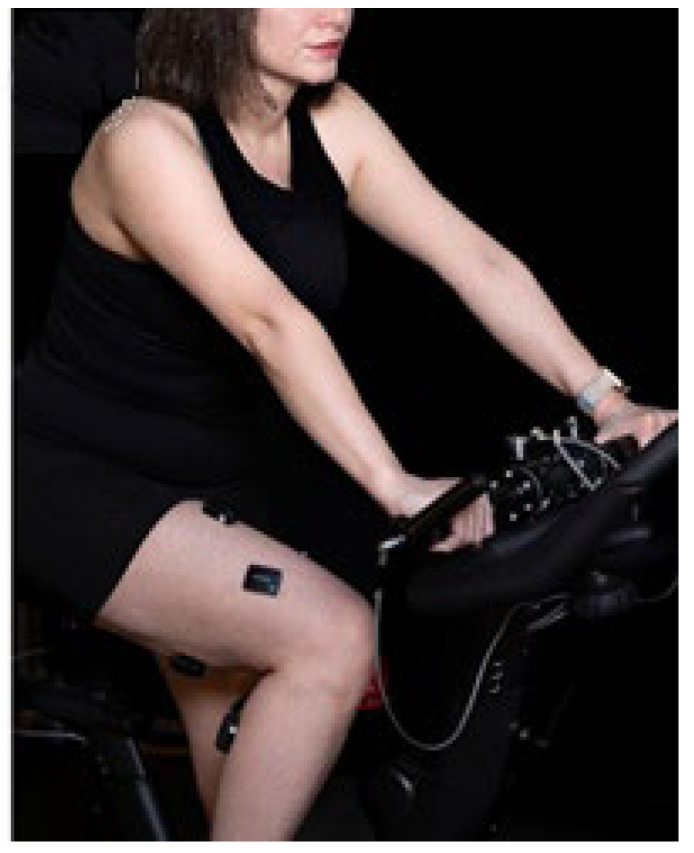
EMG sensor locations to measure activities of seven major lower-limb muscles during the pedaling tests.

EMG was recorded using a wireless 14-channel Delsys Trigno system (Delsys Inc., Natick, MA, USA), with sensors placed on seven lower-limb muscles on each leg: vastus medialis (VM), vastus lateralis (VL), rectus femoris (RF), biceps femoris (BF), soleus (Sol), gastrocnemius medialis (GM) and tibialis anterior (TA) (see Figure 2). Hip muscles, such as the gluteus maximus and hip flexors, were not analyzed due to reduced signal quality caused by EMG sensor detachment during cycling, muscle depth, and higher subcutaneous tissue [56], compromising data reliability. Sensor placement followed surface EMG for a non-invasive assessment of muscles (SENIAM) recommendations [57]. Each bipolar Trigno sensor consists of two parallel bar electrodes with an inter-electrode distance of 10 mm (center-to-center), consistent with SENIAM guidelines [58]. Before electrode placement, the skin was shaved, cleaned with alcohol, and allowed to dry to ensure low-impedance contact between the skin and the pre-gelled EMG electrodes. EMG data were sampled at 2200 Hz and synchronized with the crank angle data using the Sensix acquisition system (Sensix Inc., Poitiers, France). Raw EMG signals were band-pass filtered (20–450 Hz, fourth-order Butterworth, zero-lag), notch filtered (60 Hz), rectified, and then low-pass filtered (8 Hz, fifth-order Butterworth) to extract the linear envelope. The filtered EMG signals were subsequently analyzed to assess asymmetries between corresponding muscles on both sides.

In this study, to ensure consistency in computing NSI, EMG signals were normalized using a dynamic, task-specific reference obtained from each participant’s maximum cycling power (Pmax). Before the constant-power test, participants performed a 1-min cycling bout at Pmax, and the peak EMG value for each muscle during this phase served as its reference. EMG data from the subsequent constant-power test (70% Pmax) were expressed relative to these reference values, following recommended approaches for cycling EMG analyses [59,60,61].

### 2.5. Evaluation of Asymmetrical Lower-Limb Function

Equations (1) and (2) define the NSI calculation [30]:

(1) $$PN=\left(\frac{P-P_{min}}{P_{max}-P_{min}}\right)\;$$

(2) $$NSI\left(\%\right)=\left(\frac{{PN}_{D}-{PN}_{ND}}{\left({PN}_{D}+{PN}_{ND}\right)/2}\right)\times 100$$

Here, $P_{min}$ and $P_{max}$ represent the minimum and maximum values of P, here the EMG magnitude, within a cycle. The PN represents the normalized EMG signal, which was computed for each leg separately. The subscripts D and ND refer to the dominant and non-dominant limbs, respectively, as determined by asking participants which leg they would naturally use to kick a ball. This single-question approach is a commonly used method for determining leg dominance, particularly in cycling studies [30,62]. Moreover, Van Melick et al. [63] reported 100% agreement between self-reported and observed dominant leg (i.e., the leg identified through standardized motor tasks performed under experimental observation), supporting its validity as a reliable and straightforward measure of limb dominance. NSI values near zero indicate greater symmetry, whereas positive and negative values reflect asymmetries dominated by the dominant and non-dominant limbs, respectively.

### 2.6. Statistical Analysis

For both step incremental and constant-power tests, because participants completed the tasks in different durations, the trials were normalized by dividing the total cycling duration into five relative time points (0%, 25%, 50%, 75%, and 100% of each participant’s trial duration) to enable meaningful comparisons across participants. Statistical analyses were performed on the absolute values of each variable of interest using IBM SPSS (Version 25.0, IBM Corp., Armonk, NY, USA). The Shapiro–Wilk test was then used to assess data normality. Because the same participants contributed data at all five time points, a repeated-measures design was applied. For normally distributed variables, a repeated-measures ANOVA (RM-ANOVA) was used to examine the effect of time on NSI values for each of the seven muscles independently. When the data were not normally distributed, the Friedman test was employed as the non-parametric equivalent for within-subject comparisons. When significant effects were observed, pairwise comparisons were conducted using Bonferroni-adjusted post hoc tests (parametric) or Wilcoxon signed-rank tests (non-parametric). Effect sizes were reported as partial η^2^ for ANOVA and Kendall’s W for Friedman tests. Statistical significance was set at *p* < 0.05, and all results are presented as mean ± standard deviation (SD).

## 3. Results

**Figure 3:**
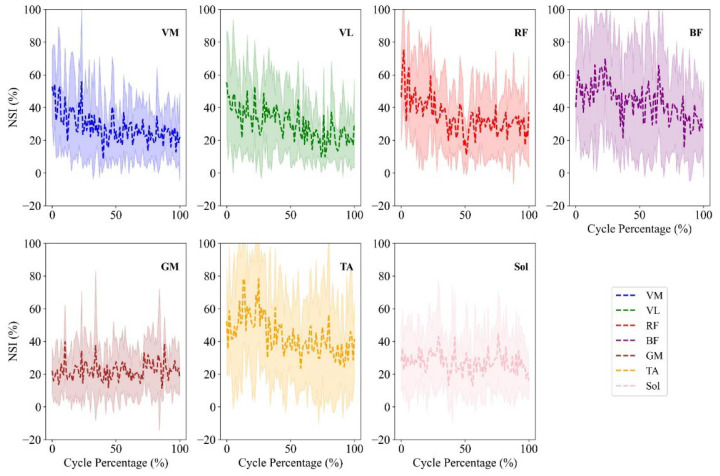
The absolute value of NSI percentage over the step incremental trial (in percentage) is shown for seven lower-limb muscles: VM, VL, RF, BF, GM, TA, and Sol. The shaded regions indicate one standard deviation above and below the mean across all participants.

**Figure 4:**
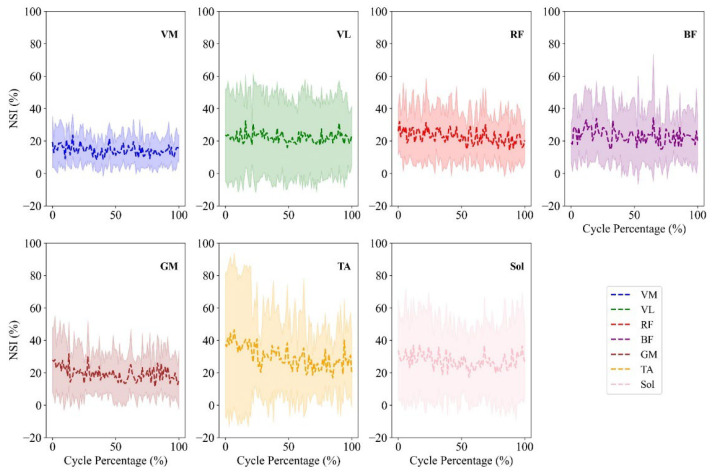
The absolute value of NSI percentage over the constant-power trial (in percentage) is shown for seven lower-limb muscles VM, VL, RF, BF, Sol, GM and TA. The shaded regions indicate one standard deviation above and below the mean across all participants.

The absolute value of the NSI percentage over the cycle percentage was analyzed in seven pairs of lower-limb muscles during the step incremental (Figure 3) and constant-power test (Figure 4). The step incremental test data from two participants were excluded due to EMG sensor detachment caused by sweating. The average and standard deviation of the absolute value of NSI for the step incremental (shaded cells) and constant-power tests for each muscle are shown Table 2.

The last column of Table 2 summarizes the statistical analysis of NSI under the step incremental and constant-power tests. The Friedman and RM-ANOVA tests revealed a significant difference in NSI values with increasing power levels in step increment data for the VM (*p* < 0.001, w = 0.285), VL (*p* = 0.002, w = 0.305), RF (*p* < 0.001, w=0.319), and BF (*p* = 0.003, w = 0.214) muscles. However, Sol (*p* = 0.123, w = 0.101), TA (*p* = 0.069, w = 0.116), and GM (*p* = 0.096, w = 0.109) did not exhibit statistically significant changes. Here, *p* denotes the *p*-value, $\eta^{2}$ denotes partial eta-squared in repeated-measures ANOVA and w is Kendall’s W value in Friedman test. In contrast, for the constant-power test, NSI values showed no significant changes over time for any of the muscles (*p* > 0.05).

## 4. Discussion

The present study investigated lower-limb muscle asymmetry in step incremental and constant-power pedaling tasks using the NSI parameter. The NSI quantitatively assesses inter-limb differences in EMG amplitude during cycling. The step incremental test provided additional insight into asymmetry compared to the constant-power condition by evaluating how NSI values evolved with increasing power output, which may reflect both differences in motor unit recruitment strategies and variations in mechanical power production. Under the step incremental condition, as cycling power increases, the NSI for the VM, VL, RF and BF muscles decreased (Figure 3 and Table 2). These muscles are primary contributors to knee and hip extension and power production during the pushdown phase of the pedal cycle [23,33,34]. This trend may reflect more uniform recruitment of motor units between limbs as task demands escalate. At higher workloads, the central nervous system likely prioritizes efficient force generation, which could naturally reduce unnecessary variability or imbalances in muscle activation [64]. This result aligns with physiological models suggesting that as intensity rises, the motor system shifts toward activating higher-threshold motor units in both limbs in a more synchronized fashion [65]. The reduction in NSI, i.e., more balanced bilateral recruitment, with increasing workload during the step incremental test is consistent with evidence of central drive symmetry and load-sharing strategies under demanding conditions [66,67]. Increased power demands necessitate greater muscle fiber recruitment, and in doing so, may reduce neuromuscular redundancy, causing more balanced activation between limbs. However, it is important to interpret these findings within the limits of the NSI metric. More specifically, while a reduction in NSI suggests reduced amplitude asymmetry, it does not imply enhanced “coordination” in a temporal or synergistic sense. Coordination, in this context, would require analysis of activation timing, phasing, or co-activation patterns, which were beyond the scope of this study.

The absence of significant NSI changes in the TA, Sol, and GM during the step incremental test may reflect their limited role in adapting to increasing workload. The TA, GM and Sol muscles contribute more to ankle stabilization and control than to forward propulsion [68]. Their smaller cross-sectional area, less force-intensive roles and inter-individual variability in signal amplitude (particularly, with differing pedaling techniques or ankle control strategies) could have contributed to the lack of consistent trends in NSI changes [65,69]. It is also worth noting that distal muscles may exhibit more variable recruitment strategies across participants, which is a known challenge in EMG studies [70]. Therefore, they may not demonstrate the same scaling in recruitment symmetry as the quadriceps muscles. Comparing these findings with previous research in walking and running has also shown that load and speed can affect inter-limb symmetry. For example, studies on gait have demonstrated that increased walking speed reduces asymmetry in ground reaction force patterns and joint angles in healthy individuals, likely due to more constrained motor control at higher outputs [71,72]. Our findings align with this literature by suggesting that increased mechanical demand, similar to higher walking speed, may promote more symmetrical muscle activation in cycling, at least in the quadriceps muscles.

In addition, while increasing power output during the step incremental test could mitigate asymmetry, the fatigue experienced during exhaustive constant-power cycling tasks would not alter the asymmetry index, as the trends remained relatively stable in Figure 4. As indicated in Table 2, none of the muscle groups demonstrated significant temporal variation in asymmetry (*p* > 0.05). Interestingly, this stable NSI profile occurred despite participants reaching task failure, indicating that neuromuscular fatigue did not significantly disrupt bilateral activation symmetry. This suggests that at constant-power pedaling conditions, the neuromuscular system may prioritize maintaining balance and a reproducible pattern of muscle activation between legs with muscle fatigue development. It should also be acknowledged that the step incremental test inherently involves both increasing intensity and the development of neuromuscular fatigue. While this study did not specifically isolate these two factors, reaching volitional exhaustion occurred in both step incremental and constant-power conditions, as it was the criterion used to terminate the test. However, the constant-power test did not show a reduction in NSI, suggesting that increasing power output, rather than fatigue alone, may have been the primary driver of the observed asymmetry changes in step incremental tests.

Previous studies have reported the NSI as a robust method for assessing the asymmetry of biomechanical parameters throughout the pedal stroke [28,29,73]. While this metric does not capture timing or coordination, it offers a useful estimate of relative inter-limb asymmetry, particularly when analyzed across different workload conditions.

From a practical standpoint, these results have implications for training and rehabilitation programs. For athletes, training protocols that incorporate progressive load increases could help promote symmetric neuromuscular engagement, potentially reducing the risk of overuse injuries associated with chronic asymmetry [1,6,74]. For the rehabilitation of unilateral conditions, such as stroke, ACL reconstruction, and joint surgeries, monitoring NSI during cycling-based exercise could offer a non-invasive method to track recovery progress and identify persistent asymmetries that might require intervention [43,44].

**Figure 5:**
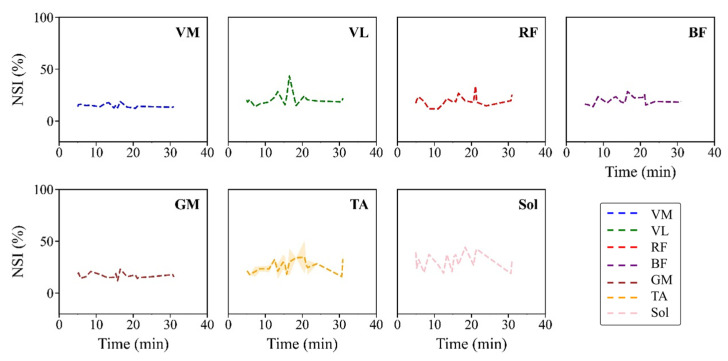
Participants’ test duration (time to task failure) and corresponding NSI at the task failure for each muscle in the constant-power test. No significant contribution was found between the NSI and test duration.

While the findings contribute to our understanding of muscle activity asymmetry during cycling, some limitations should be acknowledged. First, NSI captures only differences in EMG signal amplitude and does not account for timing, phase alignment, or co-activation between muscles. As such, the interpretation of symmetry is constrained to magnitude-based comparisons. Incorporating additional analysis techniques, such as muscle synergy decomposition, inter-muscular coherence, or time-frequency analyses, could offer a more comprehensive view of neuromuscular control strategies [69,70]. Second, while sensor placement was conducted according to SENIAM recommendations [57], and participant preparation followed best practices, surface EMG still carries inherent limitations. These include potential crosstalk between muscles and sensitivity to skin impedance or subcutaneous fat thickness. Although these limitations were consistent within each participant, future studies may consider supplementing EMG data with joint kinematics or pedal force measurements to improve interpretability [24,27]. Third, the study sample was limited to 20 recreationally active participants, with step incremental test data from two participants excluded due to sensor detachment during the test. While the remaining data provided sufficient statistical power for primary comparisons, a larger sample size may reveal subtler trends and improve generalizability. Leg dominance was assessed using a single self-report question, which is widely used [63] but remains a simplified measure; future studies could employ objective strength tests or multi-item questionnaires for a more detailed assessment. Although cadence was controlled and monitored within the 70 ± 5 RPM range, the cadence variations may still have influenced muscle activation patterns and, consequently, NSI values. This may partly explain the inter-individual variability observed [75] and suggest that future studies should further standardize cadence or include it as a covariate in statistical models. While the NSI was sensitive to workload-related changes in activation magnitude, this index does not account for temporal parameters of motor control such as onset latency, phasing, or inter-muscle coordination. Consequently, any interpretation suggesting improvements in neuromuscular coordination should be made with caution and ideally supported by complementary analyses. Moreover, our participants had no training in cycling, while the studies by Bini et al. [76] and Javaloyes et al. [25], highlighted that trained cyclists often exhibit more symmetric force and torque profiles than untrained individuals. Thus, including more experienced cyclists, as well as clinical populations (e.g., post-injury or neurological patients), would provide broader insight into how asymmetry develops and changes with physical demand. Finally, while this study focused on bilateral asymmetry, future work could also investigate intra-limb coordination patterns, particularly how muscle groups within a single limb adapt under different workloads. Combining EMG with biomechanical data (e.g., pedal force vectors, joint torques) may further elucidate the underlying control strategies [30]. Future studies should also include proximal muscles such as the gluteus maximus and hip flexors, particularly in trained cyclists, to provide a more comprehensive assessment of neuromuscular control. Figure 5. illustrating participants’ NSI (%) at task failure versus test duration shows no correlation for all seven muscles, with only minor fluctuations throughout the constant-power cycling bout. To verify this observation statistically and account for variability in test duration (reflecting individual fatigue tolerance), a linear-regression analysis was performed between NSI and task duration for each muscle. No significant relationship was found (all *p* > 0.05), and correlation coefficients were consistently low (|r| < 0.5). The VM showed a small, non-significant downward trend (r = −0.35, *p* = 0.14), and the VL a mild, non-significant upward tendency (r = +0.42, *p* = 0.07). For the remaining muscles, RF (r = +0.27, *p* = 0.26), BF (r = +0.23, *p* = 0.35), GM (r = −0.19, *p* = 0.43), TA (r = +0.28, *p* = 0.25), and Sol (r = +0.03, *p* = 0.89), no meaningful temporal changes were observed. Overall, these findings confirm that NSI remained stable across the constant-power cycling task, and that inter-individual differences in fatigue tolerance or time-to-exhaustion did not systematically bias bilateral activation symmetry.

## 5. Conclusions

This study investigated inter-limb muscle activation asymmetry in cycling using the NSI, derived from surface EMG recordings of seven lower-limb muscles under two conditions: step incremental and constant-power cycling. Under incremental-intensity cycling, as power output increased, NSI values were significantly reduced for primary power-generating muscles within the quadriceps group (VM, VL, RF). This reduction suggests that higher workloads may promote more balanced muscle activation between limbs, possibly due to increased motor unit recruitment and reduced variability under greater neuromuscular demand. Distal or secondary muscles such as the TA, Sol, and GM did not exhibit significant NSI changes, likely reflecting their limited contribution to propulsion and higher individual variability in control strategies. 

Under the constant-power condition, however, NSI values remained relatively unchanged across the pedaling cycles and across muscles, indicating consistent bilateral activation patterns during steady-state submaximal effort. These findings suggest that the NSI derived from EMG signals is sensitive to detect inter-limb muscle activation symmetry during the step incremental test; however, caution should be taken to use this measure during constant-power output tasks, possibly due to inter-subject variability. Therefore, NSI absolute value may not accurately reflect the true state of asymmetry in muscle activation or motor control. It should be noted that the NSI reflects only amplitude-based asymmetry and does not capture temporal coordination or synergistic muscle control.. 

From a practical perspective, the results of the present study highlight the potential of incorporating NSI-based monitoring into cycling programs to measure inter-limb asymmetries and monitor their temporal variations. Targeted interventions, such as unilateral strength training or cadence-controlled intervals, may help reduce asymmetries, optimize power distribution, and lower the risk of overuse injuries, thereby supporting improved cycling efficiency and performance [12,77].

A methodological consideration with the results of this study is the recognition that estimates of muscle activation via surface EMG are subject to several valid critiques. One significant concern is the insensitivity of surface EMG to small differences in exercise intensities, which can affect the precision of muscle activation measurements [78,79]. Additionally, factors such as electrode placement, skin impedance, and the signal-to-noise ratio can introduce variability in the results, further complicating the interpretation of muscle activation data. Another point of consideration is the lack of kinematic data to directly relate EMG asymmetry to joint mechanics. In addition, cadence was not strictly fixed at 70 rpm, and some fluctuations occurred, which may have influenced muscle activation patterns and NSI values. Moreover, EMG activities of the gluteus maximus muscle was not measured, limiting the assessment of proximal muscle contributions. Future work should address this by incorporating temporal and inter-muscle coordination metrics to provide a more comprehensive assessment of neuromuscular control in cycling.

## Figures and Tables

**Table 1:** The average and standard deviation (SD) of step incremental and constant-power tests.

Parameter		Mean ± SD
Duration of Test (min)	Step Incremental Test	7.67 ± 1.23
	Constant-Power Test	15.76 ± 9.32
Power output (W)	Step Incremental Test (max)	241.42 ± 65.77
	Constant-Power Test	170.48 ± 42.59
Cadence (RPM)	Step Incremental Test (max)	71 ± 8
	Constant-Power Test	73 ± 1

**Table 2:** The average NSI values of seven muscles at different stages of the trial for step incremental (shaded cells) and constant-power tests. Values are presented as mean ± one standard deviation (SD). The last column represents the statistical analysis of NSI. NSI: Normalized Symmetry Index, $\eta^{2}$: partial eta-squared, w: Kendall’s W value; Symbols indicate pairwise significance: †: compared to 0%, ‡: compared to 25%, and ⁑: compared to 50%.

NSI(%) Values in Cycling Progress (Mean ± SD)						
Trial Stage						Stat. Method
Muscle	0%	25%	50%	75%	100%	(*p*-Value) [$\eta^{2}\;or\;w$]
VM	44.84 ±12.27 ^†^	33.32 ± 16.87	24.11 ± 10.60	21.68 ± 6.47	21.26 ± 11.36 ^†^	Friedman (<0.001 *) [0.285]
	15.74 ± 8.47	15.03 ± 5.55	13.13 ± 4.19	12.62 ±6.85	14.10 ± 2.74	RM-ANOVA (0.085) [0.118]
VL	45.28 ± 26.26 ^†^	28.82 ± 19.34 ^†^	30.97 ± 15.95 ^†⁑^	17.83 ± 7.17 ^†⁑^	21.51 ± 10.15 ^†^	Friedman (=0.002 *) [0.305]
	22.98 ± 27.92	24.02 ± 30.26	19.01 ± 25.21	20.93 ± 23.65	21.58 ± 17.88	Friedman (0.100) [0.114]
RF	53.80 ± 15.84 ^†^	43.09 ±17.00 ^‡^	19.60 ± 7.91 ^†‡^	24.99 ± 14.76 ^†^	24.15 ± 11.79 ^†^	Friedman (<0.001 *) [0.319]
	26.96 ± 13.20	24.58 ± 9.68	21.57 ± 7.53	20.63 ± 7.69	19.32 ± 5.50	RM-ANOVA (0.109) [0.109]
BF	51.60 ± 19.17 ^†^	59.21 ± 26.15 ^‡^	44.71 ± 30.63	35.85 ± 19.66 ^‡^	28.91 ± 16.70 ^†‡^	Friedman (<0.003 *) [0.214]
	23.97 ± 13.94	26.02 ± 14.88	22.12 ± 13.48	22.48 ± 12.07	21.17 ± 10.72	Friedman (0.406) [0.059]
Sol	28.66 ± 11.64	32.76 ± 8.29	26.27 ± 13.30	33.86 ± 19.65	20.82 ± 11.52	Friedman (0.123) [0.101]
	29.30 ± 23.77	30.96 ± 25.25	27.10 ± 20.30	24.39 ± 22.72	30.87 ± 25.69	Friedman (0.766) [0.027]
GM	20.03 ± 9.19	25.75 ± 11.03	22.63 ±15.56	26.82 ± 12.02	22.52 ± 5.36	Friedman (0.096) [0.109]
	26.07 ± 15.48	18.38 ± 6.08	17.47 ± 10.73	19.95 ± 8.53	16.15 ± 5.90	Friedman (0.066) [0.066126]
TA	47.09 ± 14.42	64.17 ± 28.10	41.07 ± 17.27	39.68 ± 28.82	39.68 ± 28.72	Friedman (0.069) [0.120]
	39.35 ± 44.29	33.14 ± 17.36	29.77 ± 22.57	23.92 ± 15.43	26.50 ± 18.45	Friedman (0.096) [0.116]

## Data Availability

Data are unavailable due to privacy or ethical restrictions.

## Abbreviations

The following abbreviations are used in this manuscript:

BF	Biceps femoris
EMG	Electromyography
GM	Gastrocnemius medialis
NSI	Normalized symmetry index
RF	Rectus femoris
SD	Standard deviation
SENIAM	Surface EMG for a non-invasive assessment of muscles
Sol	Soleus
TA	Tibialis anterior
VL	Vastus lateralis
VM	Vastus medialis
